# A strategy to discover decoy chemokine ligands with an anti-inflammatory activity

**DOI:** 10.1038/srep14746

**Published:** 2015-10-07

**Authors:** Dayana Abboud, François Daubeuf, Quoc Tuan Do, Valérie Utard, Pascal Villa, Jacques Haiech, Dominique Bonnet, Marcel Hibert, Philippe Bernard, Jean-Luc Galzi, Nelly Frossard

**Affiliations:** 1 Biotechnologie et Signalisation Cellulaire, UMR 7242 CNRS/Université de Strasbourg, and Labex Medalis, ESBS, 300 Boulevard Sébastien Brant, 67412 Illkirch, France; 2 Laboratoire d’Innovation Thérapeutique, UMR 7200 CNRS/Université de Strasbourg, and Labex Medalis, Faculté de Pharmacie, 74 route du Rhin, 67401 Illkirch, France; 3 GreenPharma, 3 allée du Titane, 45100 Orléans, France; 4 PCBIS Plate-forme de Chimie Biologique Intégrative de Strasbourg, UMS 3286 CNRS/Université de Strasbourg, and Labex Medalis, ESBS, 300 Boulevard Sébastien Brant, 67412 Illkirch, France

## Abstract

Excessive signaling by chemokines has been associated with chronic inflammation or cancer, thus attracting substantial attention as promising therapeutic targets. Inspired by chemokine-clearing molecules shaped by pathogens to escape the immune system, we designed a generic screening assay to discover chemokine neutralizing molecules (neutraligands) and unambiguously distinguish them from molecules that block the receptor (receptor antagonists). This assay, called TRIC-r, combines time-resolved intracellular calcium recordings with pre-incubation of bioactive compounds either with the chemokine or the receptor-expressing cells. We describe here the identification of high affinity neutraligands of CCL17 and CCL22, two chemokines involved in the Th2-type of lung inflammation. The decoy molecules inhibit *in vitro* CCL17- or CCL22-induced intracellular calcium responses, CCR4 endocytosis and human T cell migration. *In vivo*, they inhibit inflammation in a murine model of asthma, in particular the recruitment of eosinophils, dendritic cells and CD4^+^T cells. Altogether, we developed a successful strategy to discover as new class of pharmacological tools to potently control cell chemotaxis *in vitro* and *in vivo*.

The chemotactic function of chemokines and their receptors is central to tissue development, inflammation, cancer and metastasis^1^. It is therefore important to understand and control the chemokine network in these diseases. Classic drug development approaches have generated receptor antagonists to modulate the chemokine system signaling. This has been proven difficult partly because of the chemokine network promiscuity^2^, but also because receptor antagonists generally have partial agonistic or inverse agonistic activities resulting into undesirable effects^3^. However, pathogens and parasites have developed original strategies to circumvent the chemokine system: these organisms express peptides^4^, soluble proteins^5^ or components mimicking the extracellular matrix^6^, that all act as decoy molecules and prevent the chemokine binding i) to their cognate receptors, and/or ii) to glycosaminoglycans, thereby collapsing extracellular density gradients and escaping host immune response. In addition, several experimental studies report on efficient chemokine neutralization by antibodies^7^,^8^, RNA oligonucleotides^9^, small molecules^10^ or decoy receptors^11^. This rationalizes the concept that chemokine blockade could be an interesting strategy to be used as an alternative to conventional receptor blockade. Chemical probes with such neutralizing activity could be useful tools to understand and/or treat chronic inflammation. CCL17 (known as Thymus and Activation-Regulated Chemokine, TARC)^12^ and CCL22 (known as Macrophage-Derived Chemokine, MDC)^13^ represent prominent examples of such effective approach. Both chemokines exert their biological effects through binding to and activating a common receptor, CCR4, which is expressed on different cell types, including T helper 2 (Th2) lymphocytes, eosinophils and dendritic cells^14^. It has also been shown that CCL17, CCL22 and CCR4 contribute to the pathogenesis of atopic diseases, like asthma and atopic dermatitis (AD)^15^,^16^, as the expression of both ligands and their receptor is enhanced in asthmatic patients and AD skin lesions^17^,^18^,^19^,^20^,^21^. However, the role of CCR4 in atopic diseases is still unclear, as some reports show that CCR4-deficient mice develop AD-like symptoms^22^ and allergic lung inflammation^23^,^24^ comparable to wild-type mice. However, in a humanized SCID murine model of allergic airway inflammation to House Dust Mite (HDM)^14^, a CCR4 neutralizing antibody administered prior to allergen challenge reduces the allergen-induced inflammation as well as the levels of Th2 cytokines in the lungs. In addition, anti-CCL17 and anti-CCL22 neutralizing antibodies^25^,^26^ as well as CCL17-deficient mice^22^ suppress airway and cutaneous inflammation. This indicates that there is a potential for molecules that could effectively and specifically neutralize CCL17 or CCL22 i) to help understand the role of CCL17 and/or CCL22, and ii) to develop new therapeutic strategies for allergic diseases.

Here, we develop a generic screening assay to discover small molecules binding to and blocking CCL17 and CCL22. We illustrate its potential by identifying i) two selective compounds neutralizing CCL17, ii) two selective compounds neutralizing CCL22, and iii) one molecule blocking both chemokines, all found in a collection of natural substances. To this end, we set up a two-step strategy that distinguishes, on a kinetic basis, ligands blocking the receptor (receptor antagonists) from those blocking the chemokine (neutraligands). A combination of biophysical, biochemical and functional studies was used to determine their chemical biological and pharmacological properties, and to show their remarkable utility as probes to study inflammatory processes *in vitro* as well as *in vivo* in a model of allergic asthma.

## Results

### Biologically active recombinant chemokines and their receptors

**Figure 1:**
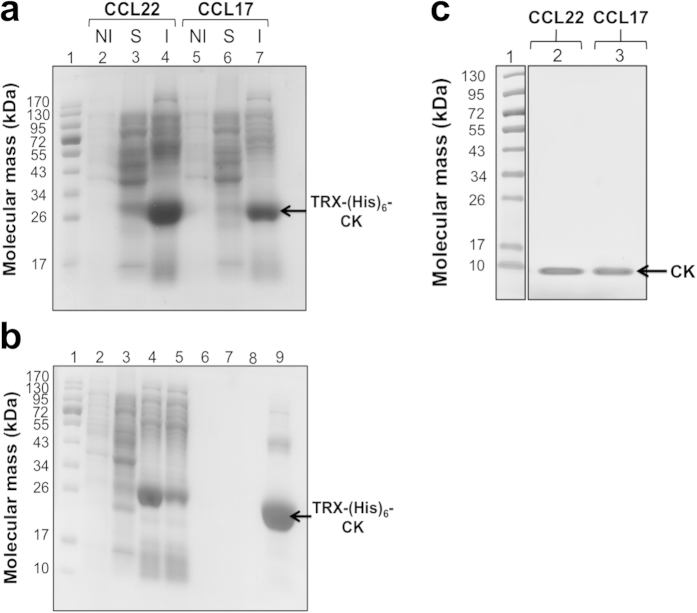
Production and purification of recombinant human CCL22 and CCL17. (**a**) *E. coli* BL21 (DE3) cells were transformed with pET32-TRX-(His)_6_-CCL17 or pET32-TRX-(His)_6_-CCL22 expression vectors. Cultures (1 ml) were collected and saved for SDS-PAGE (12%) analysis (lanes 2 and 5). Then, the production of TRX-(His)_6_ fusion CCL17 and CCL22 was induced by isopropyl-β-D-thiogalactopyranoside (IPTG) (lanes 3, 4, 6 and 7). « S » and « I » refer to the soluble (lanes 3 and 6) and insoluble (lanes 4 and 7) fractions, respectively. « NI » refers to the non-induced protein fraction (lanes 2 and 5). Lane 1: molecular weight standards. The arrow indicates the expected fused CCL17 or CCL22 band at ≈26 kDa corresponding to CCL17 or CCL22 + TRX + (His)_6_. (**b**) Purification steps of the human CCL22 on Ni^2+^-nitrilotriacetic acid (Ni-NTA) column. Samples from the « washing steps » were analysed on a 12% SDS-PAGE. Lane 1: molecular weight standards. Lane 2: non-induced fraction “NI”. Lane 3: soluble fraction “S”. Lane 4: insoluble fraction “I”. Lane 5: flow-through after incubating the insoluble fraction with the column. Lane 6: flow-through after denaturation. Lane 7: flow-through after β-cyclodextrin lavage. Lane 8: flow-through after oxidation. Lane 9: peak fractions eluted with imidazole. (**c**) Samples from enterokinase-digested chemokines were analysed on a 20% SDS-PAGE. Lane 1: molecular weight standards. Lanes 2 and 3: CCL22 and CCL17 flow-through fractions after enterokinase digestion and a second round Ni-NTA. CK: chemokine; (His)_6_: hexahistidin; TRX: thioredoxin.

Due to the need for large amounts of chemokines both for the screening and the biological studies, we produced CCL17 and CCL22. The expression was effective in *E. coli* BL21 (DE3) using the pET32 construct, which codes for the mature CCL17 or CCL22 with an N-terminal thioredoxin-hexahistidin (TRX- (His)_6_) tag followed by an enterokinase cleavage site. SDS-PAGE reveals a band present at 26 kDa for both pET32-CCL17 and pET32-CCL22 transfected *E.coli* in the insoluble fractions « I » (Fig. 1). Few or no fusion chemokine was observed in the soluble fractions « S » as well as in the non-induced « NI » samples. The insoluble fractions (as inclusion bodies) were solubilized in urea and purified on immobilized metal affinity chromatography (IMAC). Most of the chemokine CCL17 or CCL22 was retained on the Ni^2+^-nitrilotriacetic acid column by the (His)_6_ tag. Fusion chemokines were eluted and then cleaved by enterokinase to separate the TRX-His_6_ from the native functional chemokine (CCL17 or CCL22). Unwanted products (TRX, (His)_6_, uncleaved fusion chemokine, endogenous proteins) were adsorbed on a second IMAC column, leaving only pure CCL17 or CCL22 (10 kDa) in the flow-through fractions. HPLC and mass spectrometry analysis revealed that the purified chemokines exhibited the expected molecular weights and had retention times identical to those of the commercially available functional chemokines (Supplementary Fig. S1).

**Figure 2:**
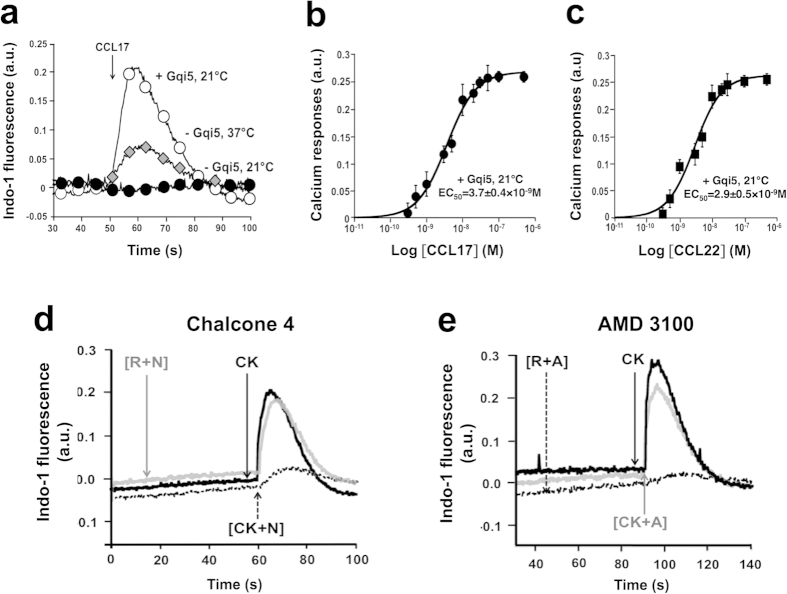
Functional assay to identify chemokine neutraligands. (**a**) Functional expression of Gqi5 in EGFP-CCR4-expressing HEK cells. Intracellular calcium responses were recorded after stimulation with 5 nM CCL17 (at t = 50 s) in EGFP-CCR4^+^HEK cells transfected with Gqi5 (⋄) at 21 °C. Gqi5-non transfected EGFP-CCR4^+^HEK cells were used as controls at 37 °C (◆) and 21 °C (•). Dose-response curves of CCL17 (**b**) and CCL22 (**c**)-induced calcium responses were performed in EGFP-CCR4^+^Gqi5^+^ HEK cells at 21 °C. Data represent the means ± SD of three independent experiments. (**d**) “Neutraligand” TRIC-r protocol. The identification of neutraligands relies on the measurement of kinetic-based calcium responses. To prove this concept, we used chalcone 4, a fully validated CXCL12 neutraligand. After stimulation with CXCL12 (at t = 60 s), EGFP-CXCR4^+^ HEK cells exhibited calcium responses (solid black line). When chalcone 4 was preincubated with CXCL12 for 1 h before addition to the cells (dotted black line), the amplitude of calcium responses was smaller than when the neutraligand was preincubated with the receptor, prior to chemokine addition (solid grey line). (**e**) “Antagonist” TRIC-r protocol. After stimulation with CXCL12 (at t = 90 s), EGFP-CXCR4^+^ HEK cells exhibited calcium responses (solid black line). When the CXCR4 antagonist (AMD3100) was preincubated with CXCL12 (solid grey line) for 1 h before addition to the cells, the amplitude of calcium responses was much greater than when the antagonist was preincubated with the receptor prior to chemokine addition (dotted black line). A: antagonist; CK: chemokine; N: neutraligand; R: receptor; the preincubation mixture is given in brackets. a.u.; arbitrary unit.

CCL17 and CCL22 are both agonists of the Gi-protein coupled receptor, CCR4. Their biological activity was tested in a calcium response assay using Human Kidney Embryonic (HEK) cells over-expressing CCR4. We found that both recombinant chemokines induced CCR4-mediated calcium responses at 37 °C, as shown for CCL17 (Fig. 2a, **grey diamonds**). These responses were specific to CCR4 expression as non-transfected cells that did not express CCR4 did not respond to CCL17 and CCL22.

### Recording of chemokine receptor-linked intracellular Ca^2+^ responses

We established an assay based on the recording of intracellular free Ca^2+^ concentration using an automated system (FlexStation 3), in order to identify new CCL17 and CCL22 neutraligands. When Gi-coupled calcium responses are very small and unreliably measured by the detector, Gqi5-, Gqtop-, Gα12- or Gα16-coupling can be used to efficiently increase the interaction of Gi-protein coupled receptors to phospholipase C (PLC) and intracellular Ca^2+^ mobilization. In this work, we generated an HEK cell line stably expressing EGFP-CCR4, and transiently transfected it with the chimeric Gqi5 protein^27^. In HEK EGFP-CCR4^+^ cells, the Gqi5 functional expression was confirmed by the measurement of large intracellular Ca^2+^ responses in response to chemokine stimulation, even at 21 °C (Fig. 2a, **white circles**). EGFP-CCR4^+^ cells that did not express Gqi5, exhibited a typically weak intracellular Ca^2+^ elevation when recorded at 37 °C (Fig. 2a, **grey diamonds**), and a non-detectable one at 21 °C (Fig. 2a, **black circles**).

We also evaluated the activity of other chemokines, CCL2 (CCR2 receptor agonist) and CCL5 (CCR1, CCR3 and CCR5 agonist)^28^ on CCR4^+^ Gqi5^+^ HEK cells (that do not express CCR2 and CCR5). We found that while CCL17 and CCL22 induced calcium fluxes, CCL2 and CCL5 did not. On the other hand, HEK cells expressing CCR2 or CCR5 responded, respectively, to CCL2 or CCL5, but not to CCL17 and CCL22 (**data not shown**). These results rule out non-specific effects that could occur in this assay.

The EC_50_ values of calcium responses induced by CCL17 and CCL22 were respectively, 3.7 ± 0.4 and 2.9 ± 0.5 nM (Fig. 2b,c), identi**c**al to those obtained with corresponding commercial chemokines. For further studies of the neutraligand inhibitory response, CCL17 and CCL22 concentrations were set at 5 nM.

The parameter describing robustness (Z’)^29^ of this calcium assay was 0.52 for CCL17 and 0.6 for CCL22 screening, allowing the use of this test to screen compound libraries.

### TRIC-r: A generic protocol to identify chemokine neutraligands

Monitoring calcium responses in a time-resolved manner allows the discrimination of compounds binding to the chemokine from those binding to the receptor. This is achieved using a pre-incubation protocol in which the tested compounds are preincubated with the receptor or with the chemokine prior to calcium monitoring. Chalcone 4 (the known CXCL12 chemokine neutraligand^10^) and AMD3100 (a CXCR4 chemokine receptor antagonist^30^) were both tested in these protocols in order to illustrate the approach, using EGFP-CXCR4^+^ HEK cells and recombinant CXCL12. When chalcone 4 was preincubated with the chemokine ([CK + N], Fig. 2d) before addition to CXCR4-expressing cells, the amplitude of calcium responses was smaller than when chalcone 4 was preincubated with the receptor ([R + N], Fig. 2d), i.e. CXCR4-expressing cells, prior to chemokine (CK) addition. By contrast, when the CXCR4 antagonist (AMD3100) was preincubated with CXCL12 ([CK + A], Fig. 2e) before addition to the cells, the amplitude of Ca^2+^ responses was much greater than when preincubated with the receptor ([R + A], Fig. 2e) prior to chemokine (CK) addition. These incubation protocols are named Time-Resolved Intracellular Calcium recordings (TRIC-r) allowing the identification of neutraligands *versus* receptor antagonists. The validity of the “TRIC-r” method here demonstrated allows an extension to a general concept of search for chemokine neutraligands.

### Automated screening of chemokine neutraligands

**Figure 3:**
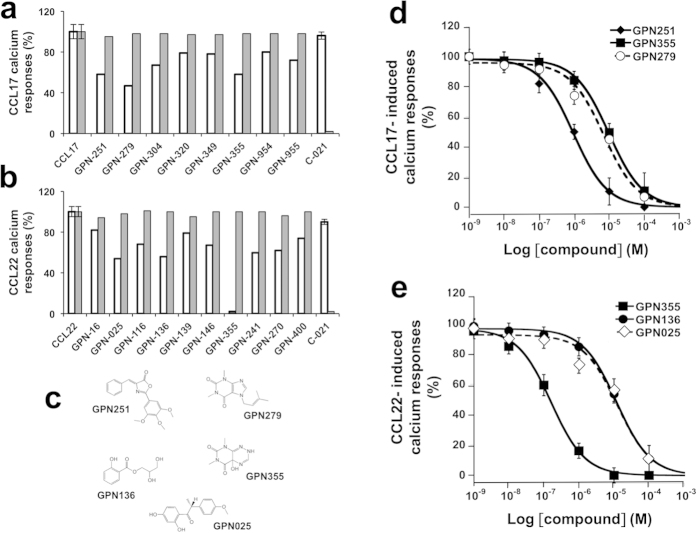
Identification of chemokine neutraligands. Compounds exhibiting a neutraligand activity, are screened on calcium responses induced by chemokines in the “neutraligand protocol”, i.e. drugs preincubated with CCL17 (**a**) or CCL22 (**b**) (5 nM, white bars) and in the “antagonist protocol” (grey bars). Results of the primary screen are expressed in percentage for compounds tested at a concentration of 2 μg/ml. C-021, the CCR4 receptor antagonist, was used as a control in both “neutraligand” and “antagonist” protocols. Molecules were considered as hits when the percentage was under 2 × SD of calcium responses induced by the chemokine alone. (**c**) Chemical structures of the 5 hit compounds. GPN251 is (*Z*)-4-benzylidene-2-(3,4,5-trimethoxyphenyl)oxazol-5(4*H*)-one. GPN279 is 1,3-dimethyl-7-(3-methylbut-2-enyl)-1H-purine-2,6(3*H*, 7*H*)-dione. GPN136 is 2,3-dihydroxypropyl 2-hydroxybenzoate. GPN355 is 4a-hydroxy-6,8-dimethyl-6,8-dihydropyrimido[5, 4-e][1, 2, 4]triazine-5,7(2*H*, 4a*H*)-dione. GPN025 is (R)-1-(2,4-dihydroxyphenyl)-2-(4-methoxyphenyl)propan-1-one. (**d**) Inhibition of CCL17-induced calcium mobilization by GPN251, GPN279 and GPN355 neutraligands. CCL17 (5 nM) was preincubated with increasing concentrations of GPN251 (◆), GPN279 (○) or GPN355 (◼) 1 h prior to addition to the EGFP-CCR4^+^Gqi5^+^ HEK cells. (**e**) Inhibition of CCL22-induced calcium mobilization by GPN025, GPN136 and GPN355. CCL22 (5 nM) was preincubated with increasing concentrations of GPN025 (•), GPN136 (◊) or GPN355 (◼) 1 h prior to addition to EGFP-CCR4^+^Gqi5^+^ HEK cells. CCR4^+^Gqi5^+^ HEK cells were loaded with the calcium-sensitive dye indo-1 AM; fluorescence intensity emitted at 401 nm and 475 nm in response to excitation at 355 nm was recorded at 21 °C. Results were analyzed by fitting the experimental curves using the Kaleidagraph software program, and the corresponding IC_50_ values were calculated. Means and SD values of triplicates were plotted.

The functional screening assay was run on a collection of c.a. 480 very diverse and natural molecules from the GreenPharma’s library (described in Supplementary Table S1). The chemicals were preincubated with CCL17 or CCL22 (neutraligand protocol) for 1 h prior to addition to the CCR4^+^cell suspension. Figure 3 shows hit compounds that reduce by more than 20% (2 × SD of control chemokine calcium responses) the amplitude of CCL17- and CCL22-calcium responses (white bars). For CCL17, the most active compounds, GPN279 and GPN251 (2 μg/ml), inhibited CCL17-induced calcium responses by 42 and 53%, respectively (Fig. 3a,c). For CCL22, GPN136 and GPN025 (2 μg/ml) blocked calcium responses by 46 and 44%, respectively (Fig. 3b,c). One compound, GPN355 (2 μg/ml) blocked both CCL17 and CCL22 calcium responses by 42 and 100%, respectively (Fig. 3a–c). None of the hit compounds did antagonize the CCR4 receptor, i.e. their preincubation with the receptor-expressing cells for 1 h did not affect the response to CCL17 or CCL22 (Fig. 3a,b, **grey bars**). Conversely, the CCR4 antagonist, C-021^31^, abolished responses when preincubated with the receptor, but not with the chemokine.

These five hit compounds were resynthesized and their dose-response curves were performed in the calcium assay. The IC_50_ values for CCL17 neutralization were 0.9 ± 0.1 μM for GPN251, 7.1 ± 1.7 μM for GPN279 and 9.6 ± 0.9 μM for GPN355 (Fig. 3d). The IC_50_ values for CCL22 neutralization were 10.6 ± 3.7 μM for GPN025, 11.6 ± 2.5 μM for GPN136, and 0.2 ± 0.0 μM for GPN355 (Fig. 3e).

Solubility studies showed that GPN279, GPN136, GPN025 and GPN355 were soluble (solubility >10 μM) in HEPES buffer and in the cell culture medium. By contrast, GPN251 had a limited solubility in HEPES buffer (0.7 μM) and was not detected as a free molecule in the culture medium. We therefore discarded GPN251 from further experiments, and continued the pharmacological characterization of the other compounds.

The cytotoxicity of GPN279, GPN136, GPN025 and GPN355 was assessed on human HUT78 T cells after 72 h of culture by measuring cell metabolic activity (Supplementary Fig. S2). The level of metabolic activity of cells treated with GPN279 or GPN136 at the highest concentration (30 μM) was similar to that of control non-treated cells, indicating no cytotoxic activity. GPN355 induced a 23.1% inhibition of HUT78 cell metabolic activity at 30 μM. In addition, a significant concentration-dependent inhibition of HUT78 cell metabolic activity was observed in the presence of GPN025 (81.3% inhibition at 30 μM), definitely confirming that GPN025 impairs HUT78 cell viability. Similar data were obtained for the human epidermal HaCat cells. Cytotoxicity of the active molecules was also tested on human monocytes in culture (Supplementary Fig. S3) using the Alamar Blue method. This confirmed the absence of cell toxicity of GPN136 and GPN279 up to 250 μM. Following these experiments, GPN025 and GPN355 were discarded from further investigations.

### Biophysical characterization of neutraligand binding

**Figure 4:**
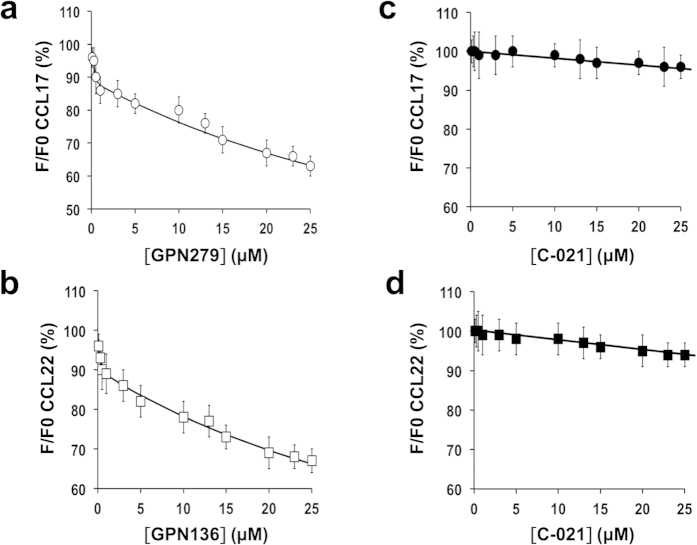
Quenching of the intrinsic tryptophan fluorescence of CCL17 and CCL22 by hit molecules. CCL17 and CCL22 were titrated with increasing concentrations (0–25 μM) of GPN279 (**a**, ○) or GPN136 (**b**,◻), respectively, and the change in tryptophan fluorescence was monitored as a function of the added ligand. As a control, the CCR4 antagonist, C-021, was tested on CCL17 (**c**,•) and CCL22 (**d**,◼). The fluorescence emission spectrum (300–400 nm) of each sample was measured at an excitation wavelength of 285 nm. The fluorescence emission spectra were corrected by subtracting the emission scan of the compound alone. DMSO had no effect on the fluorescence measurements. Titration data were normalized by dividing the measured fluorescence (F) by the initial fluorescence in the absence of the compound (F0) at 340 nm. Solid lines show the fit of the data to a second order equation: (RL)^2^ + (RL) × (-Ro-Lo-*K*_*D*_) + Ro × Lo = 0 where (RL) = ((Ro + Lo + *K*_*D*_) ± ((-Ro-Lo-*K*_*D*_)^2^-4 × Ro × Lo)^1/2^)/2 and where Ro, the concentration of CCL17 or CCL22 was set at 1.5 μM; Lo is the initial concentration of the neutraligand; *K*_*D*_is the dissociation constant of the neutraligand for the chemokine; and RL is the fractional concentration of chemokine and ligand complex. Data are means (dots) and bars are SD values of three independent experiments.

The tryptophan fluorescence technique can be used to study protein-ligand interactions since chemokines contain tryptophan residues. Several reports show that, upon binding to a protein containing tryptophan residues, the ligand modulates its intrinsic fluorescence^10^. The molecule might directly interact with the tryptophan, or bind to a site allowing protein conformational changes or modify tryptophan microenvironment, leading to altered tryptophan fluorescence. Here, we determine the fluorescence of CCL17 or CCL22 alone upon excitation of tryptophan residues at 285 nm, and detect fluorescence emission with a peak (at 340 nm) that does not show any spectral shift in the experimental conditions tested. When a fixed amount of CCL17 or CCL22 (1.5 μM) is mixed with a neutraligand (GPN279 for CCL17 and GPN136 for CCL22), a concentration-dependent decrease in tryptophan fluorescence is detected at 340 nm, reflecting binding of neutraligands to their corresponding chemokine (Fig. 4). No significant change in tryptophan fluorescence was observed with the CCR4 antagonist C-021, consistent with its lack of binding to CCL17 or CCL22. The biphasic behavior of the titration curves (discontinuity at approximately 1 μM ligand) reflects the neutraligand binding to two independent sites with each interaction producing changes in tryptophan fluorescence intensity. The binding affinity values (K_D_) of neutraligands for their respective chemokines were determined using a second order equation that was fully consistent as shown by the fit, with two categories of sites with low- and high-affinities. K_D_ values for the high-affinity binding site class were estimated as below 50.0 nM for the GPN279 and GPN136. K_D_ values for the low-affinity binding site class were 57.0 μM for GPN279 on CCL17 and 53.0 μM for GPN136 on CCL22.

### Identification of CCL17 and CCL22 residues that allow interaction with the neutraligands

**Figure 5:**
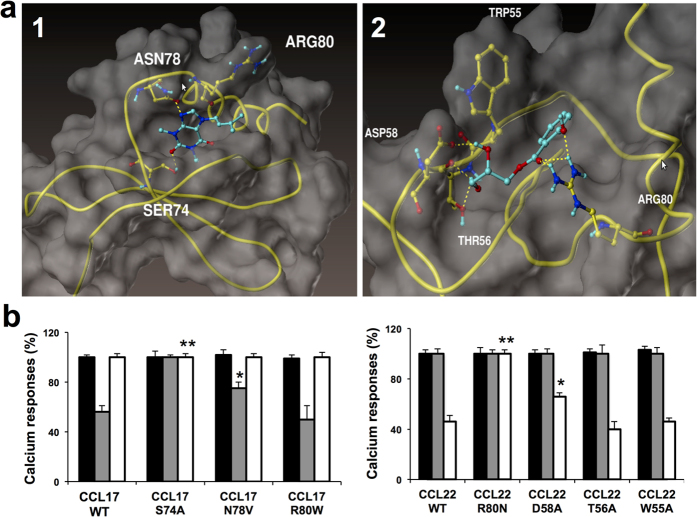
(**a**) *In silico* modeling of GPN279 and GPN136 binding to respectively CCL17 and CCL22. GPN279 docking (**a1**) highlights interaction with CCL17 Ser74, Asn78, and Arg80. GPN136 docking (**a2**) shows interaction with Asp58, Thr56, Trp55, Arg80 residues. (**b**) Effect of DMSO (black bars), GPN279 (10 μM, grey bars) and GPN136 (10 μM, white bars) on calcium responses induced by CCL17 and CCL22 mutants (5 nM). *p < 0.05; **p < 0.01 as compared to WT chemokine preincubated with the neutraligand.

In order to investigate the possible binding sites for GPN compounds on CCL17 and CCL22, we docked these ligands onto 3D structure models. CCL17 3D structure is known^32^ and that of CCL22 (which has not yet been solved) was elaborated on the basis that CCL17 and CCL22 share important sequence homologies (67% of homology) and both activate the same CCR4 receptor. As could be expected from tryptophan fluorescence studies, docking experiments revealed more than one possible interaction, suggesting the existence of two binding sites on CCL17 and CCL22. In the models, Ser74, Asn78 and Arg80 (Fig. 5a-1) from CCL17 represent plausible attachment points for GPN279, while in CCL22, a binding site for GPN136 is delineated by Trp55, Thr56, Asp58 and Arg80 (Fig. 5a-2).

To parse the contributions of these amino acids to binding of GPN279 and GPN136 binding, we expressed seven chemokine mutants, in which the residues Ser74, Asn78, Arg80 from CCL17, and Trp55, Thr56, Asp58 and Arg80 from CCL22 were substituted by Ala, Val, Trp, Ala, Ala, Ala and Asn, respectively. CCL17 and CCL22 mutants (5 nM) induced calcium responses similar to those of WT counterparts (Fig. 5b). On CCL17 S74A, GPN279 no longer acted as a neutralizing molecule. CCL17 N78V was less sensitive than WT to GPN279 (75.0 ± 6.0% instead of 56.0 ± 5.0% calcium response for WT CCL17), and CCL17 R80W was inhibited by GPN279 similarly to WT CCL17 (50.0 ± 11.0% compared to 56.0 ± 5.0%). On the same CCL17 mutants, GPN136 was totally ineffective, as expected from the absence of interaction with this chemokine. For CCL22, GPN136 no longer inhibited calcium responses induced by CCL22 R80N (100.0 ± 3.0% compared to 46.0 ± 6.0% for WT CCL22), and to a lower extent those induced by CCL22 D58A (76.0 ± 3.0%). However, GPN136 inhibited CCL22 T56A (40.0 ± 6.0%) and CCL22 W55A (46.0 ± 3.0%)-induced calcium responses similarly to CCL22 WT. GPN279 neither inhibited WT nor CCL22 mutants, confirming that this compound does not bind to CCL22. Taken together, these findings strengthen our view that Ser74 and Asn78 (in CCL17) as well as Arg80 and Asp58 (in CCL22) are likely involved in physical contacts with GPN279 and GPN136, respectively, and constitute an essential part of CCL17 and CCL22-binding sites. Noteworthy, these predicted binding sites were found in geographically distinct regions in CCL17 and CCL22.

### Generalization of TRIC-r assay for selectivity profiling

**Figure 6:**
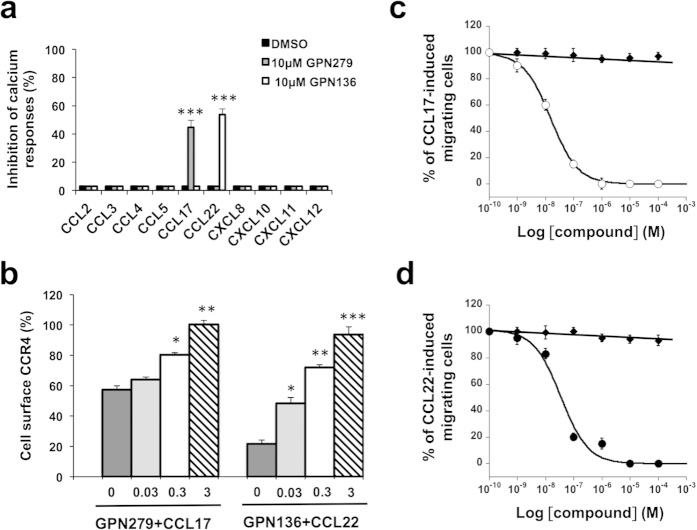
(**a**) Selectivity of CCL17 and CCL22 neutraligands towards 10 chemokines according to the “neutraligand protocol”. Inhibition by DMSO (black bars), GPN279 (grey bars) and GPN136 (white bars) (10 μM) of calcium responses triggered by either 5 nM CCL2 on EGFP-CCR2^+^ HEK cells, by 5 nM CCL3, CCL4 and CCL5 on EGFP-CCR5^+^ HEK cells, by 5 nM CCL17 and CCL22 on EGFP-CCR4^+^ HEK cells, by 5 nM CXCL8 on EGFP-CXCR2^+^ HEK cells, by 5 nM CXCL10 and CXCL11 on EGFP-CXCR3^+^ HEK cells, or by 5 nM CXCL12 on EGFP-CXCR4^+^ HEK cells. Each column represents the mean (block) ± SD (bars) of n = 3 experiments performed in triplicate. *p < 0.05; **p < 0.01; ***p < 0.005 as compared to chemokine + DMSO. (**b**) Chemokine neutraligands inhibited CCR4 endocytosis induced by CCL22 or CCL17. EGFP-CCR4^+^ HEK cells were incubated for 30 min with DMSO or chemokine (100 nM CCL22 or CCL17) in the presence or absence of test compounds. GPN279 and GPN136 were applied at concentrations of 0 μM (dark grey bars), 0.03 μM (light grey bars), 0.3 μM (white bars), and 3 μM (stripped bars). Cell surface EGFP-tagged CCR4 receptors were measured by EGFP antibody labeling and flow cytometry analysis. CCR4 internalization was expressed as a percentage of total receptor surface expression calculated using control cells incubated with DMSO (100%). Each column represents the mean (block) ± SD (bars) of n = 3 experiments performed in triplicate. *p < 0.05; **p < 0.01; ***p < 0.005 as compared to chemokine alone. (**c**,**d**) Inhibition of CCL17 and CCL22- induced CCR4^+^HUT78 T cell chemotaxis by neutraligands. Human CCR4^+^HUT78 cells were induced to migrate in the Transwell plates, where 3 nM CCL17 or CCL22 pre-incubated either with DMSO (◆) or increasing concentrations of neutraligands (GPN279 (○) for CCL17 and GPN136 (○) for CCL22) at room temperature for 1 h, and then loaded in the lower chamber. The number of migrating cells induced by a chemokine alone was set at 100% migration. The number of migrating cells in the presence of chemokine and compounds were expressed as the corresponding percentages. Results were analyzed by fitting the experimental curves (% of migrating cells *vs* log compound concentration) using the Kaleidagraph software program, and the corresponding IC_50_ values were calculated. Means and SD values of triplicates were plotted.

The selectivity of the neutraligands was evaluated on 10 different chemokines belonging to the CC and CXC subfamilies in both the neutraligand and incubation protocols. Consistent with data from Fig. 6a, GPN279 (10 μM) inhibited by 40.0% the CCL17-evoked calcium responses in EGFP-CCR4^+^HEK cells, whereas it had no effect on calcium responses induced by CCL3, CCL4, CCL5, CCL22, CXCL8, CXCL10, CXCL11 and CXCL12 chemokines on their respective receptors (**grey bars**). GPN136 (10 μM) inhibited 46.0% of the CCL22-evoked calcium responses in HEK EGFP-CCR4^+^cells, and had no effect on calcium responses induced by any other chemokine (**white bars**). GPN279 is therefore a selective CCL17 neutraligand, and GPN136 a selective neutraligand of CCL22. None of these compounds had any effect on calcium responses when preincubated with the receptors, i.e. in the “antagonist” protocol (**data not shown**).

### *In vitro* activity of chemokine neutraligands

We investigated the ability of neutraligands to inhibit CCL17 and CCL22-evoked CCR4 internalization. Receptor endocytosis was monitored on EGFP-CCR4^+^HEK cells and was quantified by flow cytometry. Confirming previous observations^33^,^34^,^35^, CCL22 induced marked CCR4 internalization (78.0 ± 3.0%), whereas CCL17 was less potent and induced approximately 43.0 ± 3.0% of receptor endocytosis at the same concentration (100 nM). Figure 6b shows that GPN279 inhibited CCL17-mediated CCR4 internalization in a concentration-dependent manner (0.03–3 μM) with half-maximal efficiency around 0.2 μM. GPN136, dose-dependently, reduced CCL22-induced internalization with half-maximal efficiency in the 0.1 μM range. Neither GPN279 nor GPN136 did alter CCR4 receptor surface level on its own (**data not shown**).

We next analyzed the effect of neutraligands on the chemotactic activity of CCL17 and CCL22 on human HUT78 T cells that endogenously express CCR4 receptor^34^,^35^,^36^. Confirming previous reports^34^, we observed that CCL17 and CCL22 induced HUT78 cell migration with a bell-shaped dose-response curve. For further studies of the neutraligand inhibitory response, CCL17 and CCL22 concentrations were set at 3 nM. GPN279 induced a concentration-dependent inhibition of the chemotactic response with an IC_50_ = 0.15 ± 0.01 nM (Fig. 6c) when preincubated for 1 h with CCL17. GPN136 also showed high potency in inhibiting CCL22-induced chemotaxis (IC_50_ = 0.34 ± 0.1 nM). Importantly, the absence of effects of the neutraligands by themselves indicates that they are devoid of chemotactic activity (Supplementary Fig. S4). This assay also reveals a drastic migration difference depending on the incubation protocol. Indeed, when the neutraligands were preincubated with the cells, as in the receptor antagonist protocol, they show no ability to inhibit CCR4^+^ HUT78 cell chemotaxis, whereas the specific CCR4 antagonist, C-021 (1 μM), inhibited 90% of T cell migration (Supplementary Fig. S4).

We also analyzed the consequences of CCL17 and CCL22 blockade on the migration of human keratinocyte HaCat cells that constitutively express CCR4^37^ in a “scratch wound” assay (Supplementary Fig. S5). While complete wound closure of scratches was seen after 72 h of treatment of HaCat cells with CCL17 and CCL22, we found that GPN279 and GPN136 (10 μM) inhibited CCL17 and CCL22-mediated wound closure by 81.4 and 85.2%, respectively.

GPN279 and GPN136 exhibit close structural similarity with respectively, theophylline^38^ (phosphodiesterase inhibitor) and aspirin (cyclooxygenase (COX) 1 inhibitor)^39^, which are known as anti-inflammatory agents. We therefore evaluated the activity of theophylline, aspirin and their derivatives (Supplementary Table S2) on CCL17- and CCL22-induced calcium responses. In contrast to GPN279 (compound 1) and GPN136 (compound 6), theophylline (compound 2) and aspirin (compound 13) had no effect on calcium responses (0.0% inhibition at 5 μM) induced by CCL17 or CCL22, respectively. We also found that GPN279 had no effect on COX1 and COX2 activity, and that GPN136 inhibited COX1 activity but only at high doses (>100 μM) (Supplementary Fig. S3). In summary, GPN279 (selective for CCL17) and GPN136 (selective for CCL22) at concentrations below 100 μM exhibit anti-inflammatory activity with a mechanism that differs from that of theophylline and aspirin.

### *In vivo* activity of chemokine neutraligands

**Figure 7:**
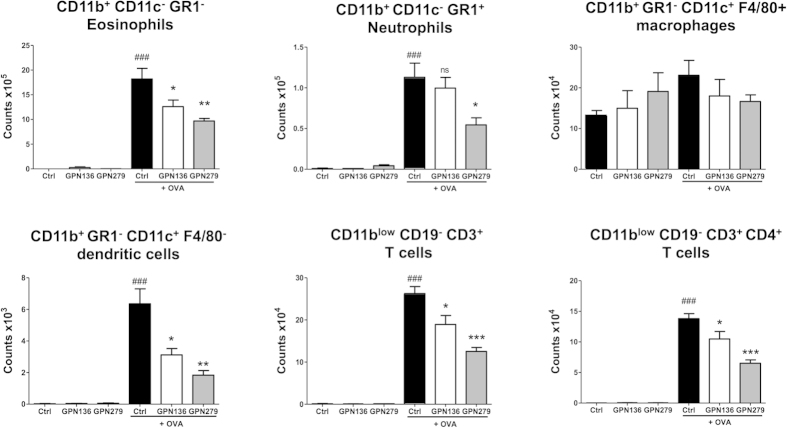
Effect of the CCL17 neutraligand (GPN279) and of the CCL22 neutraligand (GPN136) on the inflammatory cell infiltration in the bronchoalveolar lavage (BAL) in a mouse model of allergic eosinophilic airway inflammation. Balb/c mice were sensitized (i.p.) to OVA and challenged (i.n.) with OVA or saline in mice treated with solvent (black), GPN136 (white) or GPN279 (grey) administrated intraperitoneally (350 μmol/kg). The differential number of cells recovered in BAL fluid 24 h after the last challenge was counted by flow cytometry. Live leukocytes were identified as CD45^+^DAPI^−^ cells then differentiated into T cells (CD11b^low^CD19^−^CD3^+^), CD4^+^T cells (CD11b^low^CD19^−^CD3^+^CD4^+^), eosinophils (CD11b^+^CD11c^−^GR1^−^), neutrophils (CD11b^+^CD11c^−^GR1^+^), macrophages (CD11b^+^GR1^−^CD11c^+^F4/80^+^), dendritic cells (CD11b^+^GR1^−^CD11c^+^F4/80^−^).

Both CCL17 and CCL22 have been reported to participate into the development of asthma^40^. Our aim was therefore to validate the activity of our compounds in a murine model of asthma. Before doing so, we validated the neutraligand activity of GPN279 and GPN136 on calcium responses induced by the murine isoforms of CCL17 and CCL22. GPN279 inhibited mouse CCL17-evoked calcium signaling with IC_50_ = 5.2 ± 0.9 μM, and GPN136 inhibited mouse CCL22-evoked calcium responses with IC_50_ = 8.1 ± 1.5 μM (Supplementary Fig. S6), results similar to the IC_50_ values of human isoforms. We thereby confirm that GPN279 and GPN136 neutralize mouse and human CCL17 and CCL22, respectively, and may be used in a murine model of airway allergic hypereosinophilic inflammation. Mice were sensitized to- and challenged with- ovalbumin (OVA) in order to develop airway inflammation, and inflammatory parameters were measured in the absence and presence of GPN279 or GPN136. In this *in vivo* model of allergic asthma, increased inflammatory cell numbers were found in the bronchoalveolar lavage fluid (BALF). We detected in particular a massive recruitment of eosinophils (18.0 ± 1.1 × 10^5^) together with neutrophils (1.1 ± 0.1 × 10^5^), CD11c^+^ F4/80^+^macrophages (2.1 ± 0.2 × 10^5^), CD3^+^T cells (2.6 ± 0.1 × 10^5^), CD4^+^T cells (1.4 ± 0.1 × 10^5^) and CD11c^+^F4/80^−^dendritic cells (6.4 ± 0.3 × 10^3^) (Fig. 7). Systemic treatment (I.P.) with GPN279 or GPN136 (350 μmol/kg) significantly reduced the overall number of inflammatory cells, in particular of recruited eosinophils, T cells and CD4^+^T cells, and CD11c^+^F4/80^−^ dendritic cells, as compared with vehicle-treated mice (Fig. 7). While GPN279 significantly inhibited the neutrophil counts in BALF of OVA-treated mice, GPN136 did not. The number of CD11c^+^F4/80^+^macrophages was not modified in GPN279- and GPN136-treated mice compared to vehicle-treated mice. Taken together, these data strongly confirm that the decoy molecules GPN279 and GPN136 neutralizing CCL17 and CCL22, respectively, have an inhibitory effect in our *in vivo* model of allergic asthma, showing anti-inflammatory activity by reducing the accumulation of eosinophils, dendritic cells and CD4^+^T cells in the airways.

## Discussion

Emergence of targeted therapies has markedly improved the management of atopic disorders. However, treatment options for the most insidious forms of these diseases, characterized by therapeutic resistance, remain elusive. Recently, the signaling axis formed by chemokines and their receptors has been attributed a causal role in the development of atopic diseases, highlighting new opportunities for therapeutic intervention. A number of strategies have been pursued to develop chemokine receptor inhibitors, including antibodies or small molecules to block receptor signaling (antagonists). Despite these efforts, current anti-chemokine receptor therapeutics either show modest anti-inflammatory activity, or induce substantial off-target effects^2^,^41^.

Our current study shows that the novel neutraligand strategy makes the development of chemokine inhibitors a new challenging issue. Neutraligands mimic the naturally occurring decoy proteins encoded by pathogens to prevent the host inflammatory response^42^,^43^. They constitute therefore interesting tools to be added to the traditional antagonist panoply.

Toward the discovery of chemokine neutraligands, we designed a generic screening assay, which is called “Time-Resolved Intracellular Calcium recording” (TRIC-r). This functional screening is based on the kinetic measurement of chemokines-induced calcium responses. Depending on the mode of compound library preincubation either with the chemokine or with the receptor, the TRIC-r method allows a rapid and efficient identification of bioactive molecules and unambiguously identifies the target as being either the receptor or the chemokine itself. Our results validate TRIC-r as a fast, quantitative and sensitive technique that requires reasonably low amounts of biological materials and chemicals, and avoids selecting molecules that would bind to the cytokine without neutralizing its function. Even though it uses a calcium indicator, TRIC-r can be considered as a label-free technology since neither the receptor nor the chemokine need to be chemically or genetically modified. This makes TRIC-r easy to set-up and to apply to several chemokine-chemokine receptor pairs from CC- and CXC subfamilies, as illustrated here for ten of them. This could also allow the identification of neutraligands for chemokines such as CXCL14^44^ or CXCL15^45^, which have no identified receptor yet, provided that we can find cells responding to these chemokines.

Application of the TRIC-r assay to CCL17^12^ and CCL22^13^ led to the discovery of 5 structurally unrelated neutraligands, namely GPN279 and GPN251 for CCL17, GPN136 and GPN025 for CCL22, and GPN355 that targets both chemokines. Out of these molecules, GPN279 and GPN136 directly interact with chemokines, as assessed by tryptophan fluorescence alterations and by site directed mutagenesis, and thereby block cellular responses induced by chemokines, i.e. calcium responses, HUT78 chemotaxis, HaCat migration, and CCR4 receptor endocytosis.

In contrast to receptor antagonists, neutraligands do not affect the basal activity level of chemokine receptors. As do most G-protein coupled receptors, chemokine receptors exhibit constitutive activity. Pharmacological agents interacting with these receptors display a broad spectrum of activities, ranging from full agonists through partial and biased agonists to full antagonists (inverse agonists)^3^. Very few of them bind to the receptor without modifying the basal activity. Therefore, antagonists generally interfere with cell homeostasis, an effect that is absent when the chemokine itself is neutralized with antibodies or neutraligands^46^. Indeed, in the present study, the neutraligands by their own did not alter the basal level of CCR4-associated responses. This constitutes an important result since it had previously been reported that some CCR4 antagonists could act as agonists for cell chemotaxis^35^,^47^.

In addition, a recent report^34^ suggested that CCL17 and CCL22 bind to distinct CCR4 receptor conformations on leukocytes, resulting in blockade of signaling *via* one conformation but not the other. These findings represented a potential caveat against the development of CCR4 antagonists. Neutraligands may therefore be a rescue to target the CCR4 pathway.

We here propose a well-structured binding pocket for GPN136 on CCL22, and for GPN279 on CCL17, with interactions of the neutraligands with chemokine amino-acid residues. These have been evaluated by site-directed mutagenesis of Ser74, Asn78 residues in CCL17 and Arg80 and Asp58 in CCL22 preventing GPN279 and GPN136 to inhibit calcium responses, and demonstrating the validity of our proposed model.

We showed that GPN279 and GPN136 inhibit calcium responses induced by murine CCL17 and CCL22 isoforms, respectively, as efficiently as they block human chemokines. These findings are consistent with the notion that binding pockets are similar on human and mouse chemokine isoforms. In agreement, human and murine CCL22 isoforms share three identical residues (Trp55, Thr56, and Arg80) in their binding sites. Similarly, human CCL17 exhibits two residues (Asn78, Arg80) similar to those of the murine isoform (Asp78, His80). On the other hand, we notice that the negatively charged Asp58 in human CCL22 is replaced by a positively charged Lys58 in the mouse isoform, and that a polar Ser74 in human CCL17 is replaced by an apolar alanine residue in the mouse isoform. *In silico* modeling of the binding area indeed shows that amino acids surrounding the binding site are likely to exert compensatory effects, thereby accounting for the identical efficacy of GPN136 and GPN279 on human and mouse chemokines.

Since CCL17 and CCL22 are involved in the pathogenesis of atopic diseases, we finally studied the *in vivo* activity of GPN279 and GPN136 at inhibiting inflammatory cell recruitment in a mouse model of allergic asthma. Our data show reduction of eosinophil, CD4^+^T cell and dendritic cell counts by each neutraligand, reinforcing previous results upon treatment with CCL17^25^,^26^ or CCL22^25^ neutralizing antibodies in models of allergic airway inflammation.

In conclusion, we here describe a generic assay to identify neutraligands and demonstrate that chemokine neutralization offers a judicious approach for therapeutic intervention. Notably, the decoy GPN279 and GPN136 molecules validate CCL17 and CCL22 as therapeutic targets in allergy and create a path for its potential to be realized.

## Methods

### Reagents

The GreenPharma’s library (480 compounds, 2 mg/ml) was prepared in 96-well plates (Supplementary Table S1). GPN279 (purity >95%), GPN251 (purity >95%), GPN136 (purity >95%), GPN025 (purity >80%) and GPN355 (purity >90%) were synthesized by GreenPharma, and their structural identity was confirmed by mass spectrometry and ^1^H NMR analysis. C-021 and AMD3100 were purchased from Calbiochem. Human recombinant chemokines (CCL5, CCL17, CCL22, CXCL8, CXCL10, CXCL11 and CXCL12) were produced in *E.coli* BL21 (DE3), refolded and purified using Ni-NTA chromatography. The protocol is detailed in Supplementary material. Human CCL2, CCL3 and CCL4 and mouse CCL17 and CCL22 were purchased from R&D systems.

### Generation of HEK-293 cells stably expressing human chemokine receptors

Human cDNAs encoding CCR4 and CXCR3 receptors were cloned in pIRES plasmid (Clontech) while cDNAs encoding CCR2, CCR5, CXCR2 and CXCR4 receptors were cloned in pCEP4 vector (Life technologies). The receptors are cloned in frame with a signal peptide fused to enhanced green fluorescent protein (EGFP) as described previously^48^. All plasmid constructs were fully sequenced prior to transfections. Human Kidney Embryonic HEK-293 cells (ATCC) were maintained in Minimal Essential Medium (MEM, Invitrogen) supplemented with 10% FCS, penicillin (100 U/ml), streptomycin (100 μg/ml) and L-glutamine (2 mM) (Invitrogen) at 37 °C and 5% CO_2_. The cells were transfected with pIRES EGFP-CCR4 plasmid using calcium phosphate precipitation method. Stably EGFP-CCR4^+^ HEK cells were selected for 5 weeks with geneticin (G-418, 600μg/ml), and resulting cell clones were checked by fluorescence microscopy and FACS analysis. HEK cells stably expressing human CXCR2, CXCR3, CXCR4, CCR5 and CCR2 were generated as described previously^10^.

### Construction of a cell line co-expressing Gqi5 and CCR4

Human Gqi5 cDNA was produced by PCR-based site-directed mutagenesis of the last 5 amino acids of Gq into Gi using the forward primer 5′-CCTCCAGTTGAACCTGAAGGACTGCGGCCTCTTCTAACTCGAGTCTAGAGGGC-3′and the reverse primer 3′-GCCCTCTAGACTCGAGTTAGAAGAGGCCGCAGTCCTTCAGGTTCAA-CTGGAGG-5′. Gqi5 cDNA was cloned into pcDNA 3.1 plasmid (Invitrogen) by overlap extension method. EGFP-CCR4-HEK cells were transiently transfected with the plasmid encoding Gqi5. The experiments were carried out 24 h after transfection to allow for Gqi5 protein expression.

### Calcium assay

#### Cell plate preparation

EGFP-CCR4^+^Gqi5^+^HEKcells were loaded with fluorescent Ca^2+^-indicator Indo-1 AM (5μM, Interchim) for 45 min at 37 °C. The cells were detached in PBS-EDTA (5 mM) for 2 min, suspended in growth medium, pelleted by centrifugation at 320 × *g* for 5 min, and resuspended in HEPES-BSA buffer (137.5 mM NaCl, 6 mM KCl, 1.25 mM CaCl_2_, 1.25 mM MgCl_2_, 0.4 mM NaH_2_PO_4_, 5.6 mM glucose, 10 mM HEPES, BSA (0.1%, w/v), pH 7.4). Cells (100,000 cells/well, 100μl) were seeded in black clear bottom 96-well plates (Greiner), and centrifuged at 250 × *g* for 5 min.

#### Ligand plates preparation

Two plates (96-well plates, Greiner) were prepared for the screen: one containing test compounds alone in order to determine their direct agonistic activity, and another one containing test compounds mixed with CCL17 or CCL22 at room temperature for 1 h (neutraligand protocol). The chemokines and test compounds were prepared at a 3× concentration, while digitonin was prepared at a 4× concentration. The CCR4 antagonist, C-021 served as control in the experiment. In the antagonist protocol, compounds were added to the cell plate (one compound per well) and incubated with cells for 30 min at room temperature, while the agonist (CCL17 or CCL22) was added to the ligand plate only.

#### Recording calcium responses in Indo-1 loaded CCR4^+^ Gqi5^+^ HEK cells

Dye loaded cells were placed in the FlexStation 3 (Molecular Devices). Calcium mobilization was measured at 401 and 475 nm emission fluorescence after excitation at 355 nm. For the primary screening, the ligands (50μl of chemokine mixed with DMSO or test compounds) were added to the cells at 40 s, followed by digitonin addition (50μl) at 100 s to the cells. Molecules were screened at a final concentration of 2μg/ml with 5 nM CCL17 or CCL22. DMSO final concentration did not exceed 1% on cells. Raw fluorescence data (at 401 and 475 nm) were exported and the ratio 401/475 was calculated. The calcium response values (height of peak response over baseline) were determined. Hits were selected by comparing the response amplitude at the time of the chemokine addition ± test compound. The Z’ factor^29^ was calculated according to the following equation: Z’ = 1-(3SD_+_ + 3SD_−_)/|Ave_+_ − Ave_−_|, where SD_+_ and Ave_+_ are respectively the standard deviation and the mean value of the positive controls (chemokine + DMSO), while SD_−_ and Ave_−_ are respectively the standard deviation and the mean value of the negative controls (HEPES-BSA (0.1%) mixed with DMSO, or cells mixed with C-021). Hit molecules were confirmed by performing concentration-response curves and their half maximal inhibitory concentration (IC_50_) values were calculated using the Kaleidagraph software.

### Tryptophan fluorescence assay

Fluorescence measurements were performed on a spectrofluorometer (JobinYvon/Spex) in a quartz microcuvette at 20 °C. CCL17 and CCL22 (1.5 μM in HEPES buffer without BSA) were excited at 285 nm and emission spectra were recorded (300–400 nm). Initial fluorescence intensities (F0) of CCL17 or CCL22 alone were acquired at 340 nm, followed by subsequent readings (relative fluorescence, F) after sequential addition of 1μl of compounds (GPN279 or GPN136 or C-021 in DMSO) or DMSO alone. The fluorescence emission spectra were corrected by subtracting the emission scan of the compounds alone and data analysis was carried out with fluorescence intensities detected at 340 nm.

### Molecular modeling studies

Molecular modeling was performed with Sybyl-X 2.1 from Tripos (now Certara) except otherwise mentioned.

#### CCL22 Homology modeling

CCL17 was chosen as a template structure to build CCL22 model. Human CCL17 structure is available in PDB (PDB code: 1NR4). Human CCL17 and CCL22 primary sequences were retrieved from www.uniprot.org website (Accession number: Q92583 - CCL17_HUMAN & O00626 -CCL22_HUMAN respectively). ClustalW^49^ as implemented on the server NPS@^50^ was used to align both sequences. Then the module Orchestrar of the Sybyl package was used to build the CCL22 3D structure based on CCL17 (parameters are default one: find and build the conserved regions of chemokines, add loops, model side-chains and minimize the internal energy of the modeled structure to release constraints).

#### Docking studies

Docking studies were performed with Surflex-dock^51^ as implemented in the Sybyl package. We used the default parameters and docking mode “GeomX” to obtain 20 conformations for each neutraligand. The “protomol” (which defines the docking site) was defined according to the identified binding sites.

### EGFP-CCR4 receptor internalization

EGFP-CCR4^+^ HEK cells (1 × 10^6^ cells/ml) were suspended in HEPES-BSA (0.1%), and were treated with DMSO or with 100 nM CCL17 or CCL22 in the presence of increasing concentrations of GPN279 and GPN136 for 30 min at 37 °C. CCR4 levels were evaluated by flow cytometry (FACSCalibur, BD biosciences) using cell surface labeling of EGFP with monoclonal mouse anti-GFP (Roche Applied Science; 1:100 dilution) as primary antibody and R-phycoerythrin conjugated AffiniPure F (ab’)_2_ fragment goat anti-mouse (Immunotech; 1/100) as secondary antibody.

### Chemotaxis assay

The T- cell line, HUT78 (ATCC), was cultured in RPMI 1640 supplemented with streptomycin, penicillin and 10% FCS without phenol red. Cell migration was carried out in RPMI1640-BSA (0.1%) using Transwell 96-well plates (Corning Costar, 5 μm pore size, polycarbonate membranes). CCR4^+^ HUT78 cells were labeled with calcein AM (Molecular Probe, Invitrogen), re-suspended at 2 × 10^6^/ml in RPMI 1640-BSA (0.1%) and placed in the upper wells of the Transwell plate. Lower wells contained the same medium diluted with DMSO or chemokine (3 nM CCL17 or CCL22). In inhibition studies, chemokines were pre-incubated with various concentrations of compounds (GPN279 or GPN136) in the lower compartment. After a 2 h incubation at 37 °C and 5%CO_2_, the plates were read with an excitation wavelength of 485 nm and an emission wavelength of 535 nm.

### Mouse model of allergic eosinophilic airway inflammation (21 days)

Nine week-old male Balb/c mice (Janvier) were sensitized on days 1 and 7 by intraperitoneal injection of ovalbumin (OVA, 50μg, grade V, Sigma-Aldrich) adsorbed on aluminium hydroxide (2 mg, Sigma-Aldrich) in PBS. Mice were challenged intranasally with OVA (10 μg) in 25μl of saline on days 18–21. Control mice received intranasal administrations of saline alone. Mice received GPN279 or GPN136 (350 μmol/Kg) in suspension in 1% carboxymethylcellulose (CMC, Sigma-Aldrich) or vehicle (1% CMC) administered intraperitoneally 2 h before each saline or OVA challenge. Animal experimentation was approved by the local ethics committee (Comité Régional d’Ethique en Matière d’Expérimentation Animale de Strasbourg CREMEAS) and carried out in accordance with the approved guidelines. Collection of the bronchoalveolar lavage fluid (BALF) was performed 24 h after the last OVA challenge as described previously^52^. Cell counts were assessed by flow cytometry (LSRII® cytometer, BD bioscience). BAL cells were added with FCblock (5 μl, 553142, BD bioscience) in a black microplate, incubated for 20 min at room temperature. Then, marker antibodies were added: CD11c-FITC (557400, BD bioscience), Gr-1-Pe-eFluor610 (61-5931-82, eBioscience), CD11b-APC-Cy7 (557657, BD bioscience), CD45-AlexaFluor700 (103128, BioLegend), CD3-BV605 (564009, BD bioscience), CD4-PerCP-Cy5.5 (45-0042-82, eBioscience), F4/80-PE (12-4801-82, eBioscience). Antibodies were incubated for 30 min before DAPI (5 μl, BD bioscience) addition, and flow cytometry was performed immediately.

### Statistics

Data from *in vitro* studies were presented as means ± standard deviation (SD), and were analyzed using unpaired two-tailed Student’s *t* tests (StatView software). *p < 0.05; **p < 0.01; ***p < 0.005. Data from *in vivo* studies were presented as means ± standard error of the mean (SEM) (n = 3–6/group). Data were statistically analyzed by one-way ANOVA followed by Tukey’s post-test conducted with Prism software (GraphPad). ## *p* ≤ 0.01; ### *p* ≤ 0.001 *vs* solvent group, and *p ≤ 0.05; **p ≤ 0.01; ***p ≤ 0.001 *vs* OVA group.

## Additional Information

**How to cite this article**: Abboud, D. *et al*. A strategy to discover decoy chemokine ligands with an anti-inflammatory activity. *Sci. Rep*. **5**, 14746; doi: 10.1038/srep14746 (2015).

## Supplementary Material

Supplementary Information
