# Supplementary material for: A strategy to discover decoy chemokine ligands with an anti-inflammatory activity
Source: Sci Rep. 2015 Oct 7;5:14746. doi: 10.1038/srep14746 (PMC4595804; doi:10.1038/srep14746)
Supplement: Supplementary Information [file srep14746-s1.pdf]

A strategy to discover decoy chemokine ligands with an anti-inflammatory activity

Dayana Abboud[1], François Daubeuf[2], Quoc Tuan Do[3], Valérie Utard[1], Pascal Villa[4], Jacques Haiech[2], Dominique Bonnet[2], Marcel Hibert[2], Philippe Bernard[3], Jean-Luc Galzi[1#*] & Nelly Frossard[2#]

[1]Biotechnologie et Signalisation Cellulaire, UMR 7242 CNRS/Université de Strasbourg, and Labex Medalis, ESBS, 300 Boulevard Sébastien Brant, 67412 Illkirch, France

[2]Laboratoire d'Innovation Thérapeutique, UMR 7200 CNRS/Université de Strasbourg, and Labex Medalis, Faculté de Pharmacie, 74 route du Rhin, 67401 Illkirch, France

[3]GreenPharma, 3 allée du Titane, 45100 Orléans, France

[4]PCBIS Plate-forme de Chimie Biologique Intégrative de Strasbourg, UMS 3286 CNRS/Université de Strasbourg, and Labex Medalis, ESBS, 300 Boulevard Sébastien Brant, 67412 Illkirch, France

[#]These authors contributed equally to the work

*Corresponding author:

Jean-Luc Galzi, UMR 7242 CNRS/Université de Strasbourg, ESBS, 300 Boulevard Sébastien Brant, 67412 Illkirch, France, Tel +33 368 85 47 59, Fax +33 368 85 46 83, galzi@unistra.fr

Supplementary information

Supplementary material

Reagents

- GPN279 RMN ^{1}H (400 MHz, $CDCl_3$): d 1.74 (s, 6H, 2 CH_3); 3.34 (s, 3H, NCH_3); 3.51 (s, 3H, NCH_3); 4.86 (d, 2H, J = 7.25 Hz, CH_2); 5.38 (m, 1H, CH); 7.49 (s, 1H, CH). GPN251 RMN ^{1}H (400 MHz, $CDCl_3$): d 8.4 (m, 2H); 7.5 (m, 3H); 7.4 (s, 2H); 7.22 (m, 1H); 4.00 (s, 6H); 3.98 (s, 3H).
- GPN136 RMN ^{1}H (250 MHz, $CDCl_3$): d 2.61 (sl, 1H, OH); 3.07 (sl, 1H, OH); 3.68 – 3.78 (m, 2H, CH_2); 4.05 – 4.15 (m, 1H, CH); 4.39 – 4.51 (m, 2H, CH_2); 6.88 (td, 1H, *J* = 1.0 et 7.6 Hz, CH Ar); 6.98 (dd, 1H, *J* = 0.9 et 8.4 Hz, CH Ar); 7.46 (td, 1H, *J* = 1.7 et 7.7 Hz, CH Ar); 7.84 (dd, 1H, *J* = 1.7 et 8.0 Hz, CH Ar); 10.57 (s, 1H, OH).
- GPN025 RMN ^{1}H (400 MHz, $CDCl_3$): d 12.91 (s, 1H); 7.72 (d, *J* = 12.0 Hz, 1H); 7.28-7.21 (m, 2H); 6.87 (d, *J* = 12 Hz, 2H); 6.35-6.29 (m, 2H); 5.59 (s, 1H); 4.60 (q, *J* = 8 Hz, 1H); 3.79 (s, 3H); 1.52 (d, *J* = 8 Hz, 3H).
- GPN355 NMR ^{1}H (300 MHz, DMSO-d_6): d 9.72 (s, 1H, CH); 3.75 (s, 3H, CH_3); 3.39 (s, 3H, CH_3).

Recombinant human CCL17 and CCL22 production and purification

Chemokine expression. cDNAs encoding wild type CCL17, CCL22 and CXCL11 were subcloned into the pET32 vector (Novagen) containing a thioredoxin-hexahistidin (TRX-$(His)_6$) tag and an enterokinase protease site, so that these chemokines were fused to TRX with the $(His)_6$ tag. cDNAs encoding mutant CCL17 S51A, CCL17 N55V, CCL17 R57W and CCL22 R80N were synthesized by GeneArt (Life technologies) and were cloned into the pET32 vector (Novagen). cDNAs encoding wild type CXCL8, CXCL10, CXCL12 and CCL5 were cloned into the pET28 vector (Novagen) in order to fuse these chemokines to $(His)_6$ tag. All constructed plasmids were verified by DNA sequencing. The pET plasmids were transformed into competent *E. coli* BL21 (DE3) strains (Novagen). Non-transformed cells

were used as controls. Transformed cells were spread on Luria-Bertani LB agar (Euromedex) plates containing 100µg/ml of antibiotic (ampicillin (Euromedex) for pET32 CCL17, CCL22 and CXCL11, or kanamycin (Euromedex) for pET28 CXCL12, CXCL8, CXCL10 and CCL5). Plates were incubated overnight at 37°C. A single colony from the plate was inoculated in 2ml of LB broth (AthenaES) medium supplemented with the same antibiotics (ampicillin or kanamycin). Cultures were grown at 37°C overnight under vigorous agitation on a horizontal shaker. The next morning, the same medium was inoculated to the saturated overnight culture in a baffled-bottom flask. Cells were grown at 37°C for 2h under vigorous agitation. Once OD_{600} reached 0.6, expression of chemokines was induced by the addition of isopropyl- β-D-thiogalactopyranoside (IPTG) (Uptima) to a final concentration of 1mM. After 4h at 37°C, cells were harvested from liquid culture after centrifugation at 2,000×*g* for 15min. One ml of the non induced fraction was collected before the IPTG addition as control.

CCL17 and CCL22 purification. The cell pellet was suspended in BugBuster reagent (Novagen), and the cell suspension was incubated on a rotating mixer for 1h at room temperature. The supernatant (protein soluble fraction) was discarded after ultracentrifugation at 15,000×*g* for 15min at room temperature, and analysed by Sodium Dodecyl Sulfate Polyacrylamide Gel Electrophoresis (SDS-PAGE). The pellet containing the inclusion bodies including CCL17 or CCL22 was solubilized in the « inclusion bodies extraction Buffer » [100mM 2-amino-2-hydroxymethyl-1,3-propanediol hydrochloride (Tris-HCl) (Sigma-Aldrich) pH 8.3, 300mM sodium chloride (NaCl) (Sigma-Aldrich), 8M urea (Sigma-Aldrich)] and incubated on a rotating mixer for 24h at room temperature. The lysate was centrifuged at 15,000×*g* for 15min at room temperature, and the supernatant (protein insoluble fraction) was collected, analysed by SDS-PAGE and loaded onto an equilibrated nickel-nitrilotriacetic acid (Ni-NTA) column (Jena Bioscience) for a 1h incubation at 4°C. The bottom cap was removed and the column flow-through was collected and saved for SDS-PAGE analysis. The column was sequentially washed with the following buffers: « detergent buffer » [(20mM Tris-HCl pH 8.3, 300mM NaCl, 1% TritonX-100 (Sigma-Aldrich), 10mM β-

mercaptoethanol (Sigma-Aldrich)], followed by « β-cyclodextrin buffer » [(20mM Tris-HCl pH 8.3, 300mM NaCl, 5mM β-cyclodextrin (Sigma-Aldrich)], « oxidation buffer » [(20mM Tris-HCl pH 8.3, 300mM NaCl, 5mM β-cyclodextrin, 1mM reduced glutathione (Calbiochem), 1mM oxidized glutathione (Calbiochem)]. The column was incubated with the « oxidation buffer » for 24h at 4°C. All « wash fractions » were collected and saved for SDS-PAGE analysis. Recombinant CCL17 and CCL22 fusion proteins were refolded using oxidizing and reducing glutathione, because a large portion of these chemokines cannot fold properly and tends to precipitate into inclusion bodies when expressed in bacteria. The fusion protein (CCL17 or CCL22) was eluted with the « elution buffer » (20mM Tris-HCl pH 8.3, 300mM NaCl, 500mM imidazole (Sigma-Aldrich), 1.9M urea). The eluted fractions were collected and analysed by SDS-PAGE. Fractions containing the fusion protein were pooled and added to an equilibrated PD-10 desalting column (GE Healthcare) in order to desalt and exchange the sample buffer before digestion. The enterokinase (EnterokinaseMax^{TM}, Invitrogen) was added to this solution in order to remove the TRX-$(His)_6$ tag. Cleavage was performed for 16h at 22°C. The protein mixture after enterokinase cleavage was applied to a second round of Ni-NTA column. The pure cleaved chemokine (CCL17 or CCL22) passes through the Ni-NTA column while the residual undigested fusion protein, TRX-His_6 tag, the enterokinase protease and any contaminants are retained. The fractions containing the cleaved chemokine (CCL17 or CCL22) were collected and analysed by SDS-PAGE. Further purification step was carried out by gel filtration chromatography (Superdex^{TM} 75 10/300 GL, GE Healthcare) and the fractions containing the monomeric form of cleaved chemokine (CCL17 or CCL22) were collected, analysed by SDS-PAGE and concentrated on Amicon centrifugal filter (Millipore) with molecular weight cut-off at 3-4 kDa. CCL17 or CCL22 concentration was determined by optical density at 280nm. To ensure the identity of CCL17 and CCL22, high-performance liquid chromatography (HPLC) with tandem mass spectrometric (LC/MS/MS) detection method was used to validate their molecular weights and their correct folding compared to the commercial chemokines (purchased from Almac)

and the denaturated chemokines (disulfite bonds reduced by β-mercaptoethanol). The chemokine activity was then, tested in the *in vitro* assays.

On-column refolding and purification of CCL5, CXCL8, CXCL10, CXCL11, CXCL12, CCL17 mutants (S74A, N78V, R80W) and CCL22 mutants (R80N, D58A, T56A, W55A) were performed in a similar fashion as for CCL17 and CCL22.

Solubility measurements

The solubility of GPN279, GPN251, GPN136, GPN025 and GPN355 was measured in the three different buffers used in the *in vitro* assays (HEPES buffer and complete cell culture medium). Standard stock solutions of compounds (10mM) were prepared in DMSO, and the molecules were dissolved at a concentration of 10μM in each of the three buffers. The samples were shaken for 1h at room temperature, and centrifuged at 15,000×*g*. The compounds concentration in the supernatant solutions was determined using RP-HPLC (Gilson) technique. The measurements were done with a UV detector set at 230nm. The measurements were carried out at room temperature using a C18 column (26μm, 50×4.6mm, Phenomenex). The aqueous mobile phase contained 0.05% trifluoroacetic acid (solvent A, Sigma-Aldrich). The organic phase was HPLC grade acetonitrile (Sigma-Aldrich CHROMASOLV) (solvent B). The mobile phase flow rate was 2ml/min, and the following program was applied for the elution: 0–0.1min, 5% B; 0.1–2.6min, 5–95% B; 2.6–3.1min, 95% B; 3.1–3.3min, 95–5% B; and 3.3–6min, 5% B. To establish external calibration curves, increasing concentrations of each compound were prepared from standard stock solutions. The chromatograms were recorded by injecting 20μl of each standard solution, and the calibration curves were plotted using peak areas.

WST-1 metabolic activity assay

The colorimetric WST-1 cell metabolic assay (Ozyme) was used to assess the cytotoxic effect of chemokine neutraligands. This is a cell viability test based on the enzymatic cleavage by the mitochondrial succinate-tetrazolium reductase of a tetrazolium salt, WST-1 (4-[3-(4-Iodophenyl)-2-(4-nitrophenyl)-2H-5-tetrazolio]-1,3 benzene disulfonate), into a water-

soluble formazan dye which can be detected by absorbance at 420-480nm. HUT78 T cells (or HaCat cells) were seeded on 24-well plates (200,000 cells per well) and were incubated with the test compound (GPN279, GPN136, GPN025 or GPN355) or DMSO (final concentration ≤1% on cells). The background control was reserved as one well containing the culture medium only. The plates were incubated for 72h at 37°C. Three days later, the medium containing the neutraligands was removed, and WST-1 reagent (1:10 final dilution) (Ozyme) was added to each well for a 2h incubation at 37°C as the manufacturer's recommendation. The plates were shaken and absorbance was measured at 450nm (reference wavelength 690nm) against the background control, using a multiwell plate reader (EnVision, Perkin Elmer). Assays were performed as three independent replicates.

In vitro cell migration assay (scratch wound test)

The immortalized human keratinocytes cell line HaCat was purchased from DKFZ (Heidelberg, Germany) and was cultured in Dulbecco's Modified Eagle Medium (DMEM) (Invitrogen) supplemented with 10% fetal bovine serum (FBS) (Gibco-BRL), 100U/ml penicillin (Invitrogen), 100µg/ml streptomycin (Invitrogen) and 2mM L-glutamine (Invitrogen) at 37°C and 5% CO_2. The cells received fresh medium every other day and were subcultured every 10 days. HaCat cells were seeded in a 24-well plate (ESSEN Bioscience) at a density of 200,000 cells per well, and were allowed to grow until confluence. A scratch wound along the cell monolayer was created using the Essen woundmaker™. Cells were washed twice with PBS to remove detached cells, and were treated with serum-free culture medium in the absence or presence of 100ng/ml chemokine (CCL22 or CCL17) preincubated with DMSO or with the test compound (10µM GPN279 or GPN136). The molecules were also tested alone without the chemokine. The final concentration of DMSO on cells did not exceed 1%. One µg/ml of mitomycin C (Sigma-Aldrich) was added throughout the experiment in order to block cell proliferation. Once treated, the cells were immediately placed into the IncuCyte™ live-cell imaging, and the cell migration onto the wounded area was examined for 72h. Phase-contrast images acquired for each sample were captured every 2h (objective lens x10), and

the wound closure was determined as the difference between wound width at times 0 and 72h. This experiment was repeated three times.

Preparation of monocytes

Human primary monocytes were isolated from buffy coats of healthy human blood donors and seeded in 24-well plates (500,000 cells/ml in 1ml) for COX-1/2 activity or 96-well plates for cell viability experiments.

Evaluation of cytotoxicity of GPN279 and GPN136 in primary human monocytes

Cells were seeded in 96 well plates (5,000 cells/well in 200µl) for cytotoxicity testing (Alamar Blue). Cells were incubated with LPS and the 2 compounds (GPN279 and GPN136, 5 doses). After 24h, cytotoxicity was evaluated in the wells using the Alamar Blue method. NaF (250µg/ml) was used as a control in this experiment.

Cyclooxygenase (COX) activity

COX1 mediated prostaglandin E2 (PGE2) release. Monocytes were treated with arachidonic acid (AA; 15µM) in the absence or presence of GPN279 or GPN136 (100µM). Supernatants were then collected for the determination of PGE2 by enzyme immunoassay (EIA) (Cayman).

COX2 mediated prostaglandin E2 (PGE2) release. Monocytes were incubated with LPS (10U/ml) for 24h to induce COX2 protein synthesis. After renewal of medium (serum free), the cells were treated with or without GPN279 or GPN136 (100µM), which were added 15min before AA treatment. The activity of COX2 was determined by measuring PGE2 (Cayman) in the supernatants by EIA. Aspirin (10µM) and diclofenac (10µM) were used as control inhibitors.

Supplementary figures

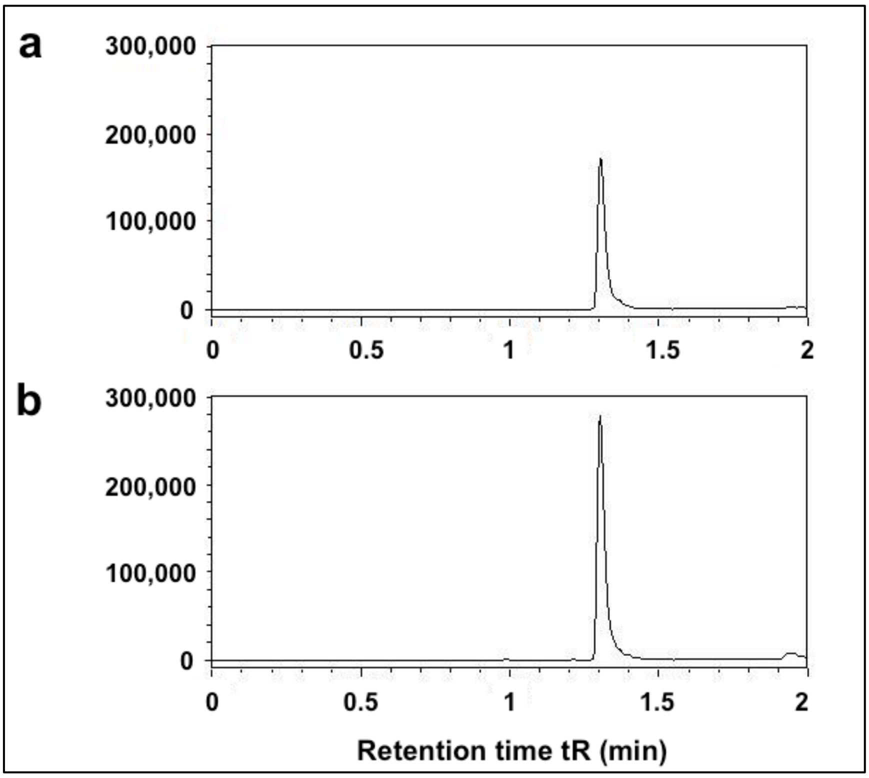

Supplementary Figure S1. The high-performance liquid chromatography (HPLC) with tandem mass spectrometric (LC/MS/MS) detection method reveals that the purified recombinant human CCL17 (**a**, 0.6µM) exhibits the expected molecular weights (8,079 Da) with an m/z ratio of 898.5 and identical retention times (1.3min) as the commercial CCL17 chemokine (**b**, 1µM, Almac).

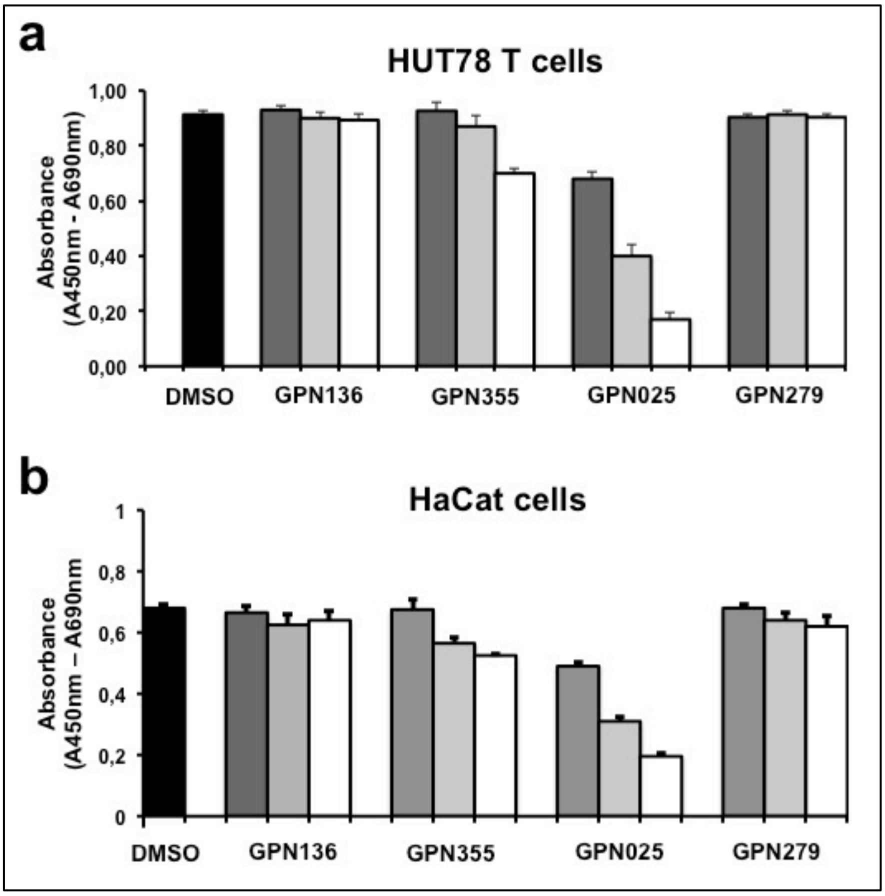

Supplementary Figure S2. *In vitro* cytotoxic activity of chemokine neutraligands on human HUT78 T cells (**a**) and HaCat cells (**b**). Cell cultures were treated with increasing concentrations [0.3µM (dark grey bars), 10µM (light grey bars), and 30µM (white bars)] of GPN136, GPN355, GPN025 or GPN279 *vs* DMSO (black bars). The metabolic activity was determined 72h after neutraligand addition. Results represent the absorbance (measured at 450 and 690nm) means±SD of three independent experiments performed in triplicate.

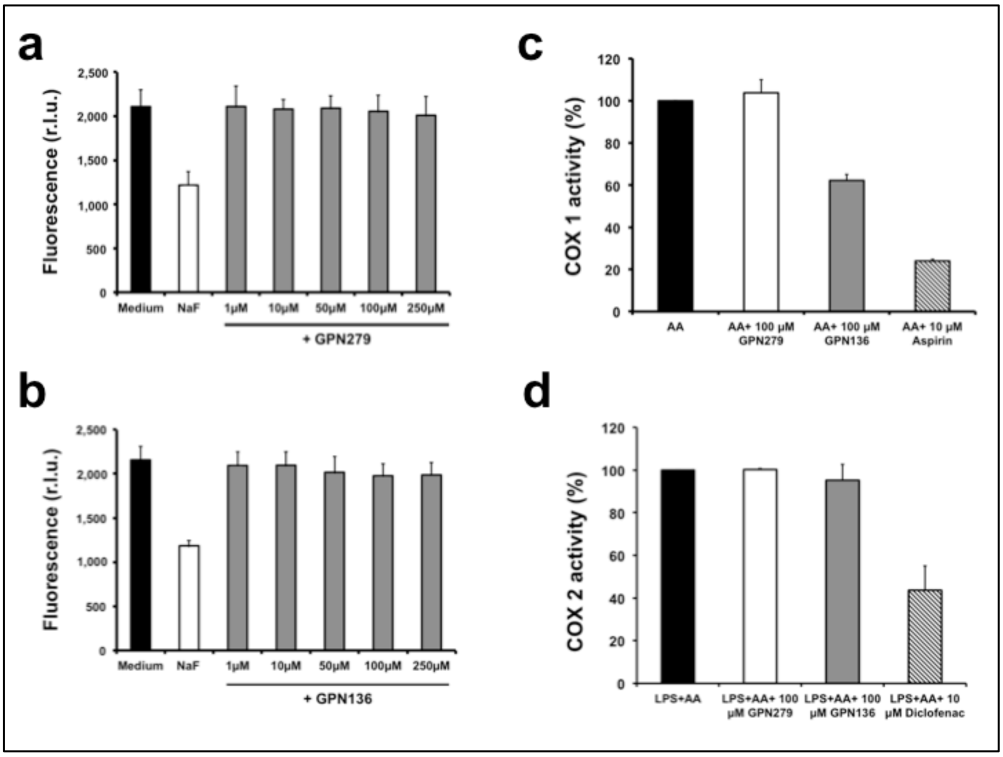

Supplementary Figure S3. Activity of GPN279 and GPN136 on cyclooxygenases (COX) 1 and 2. Effect of GPN279 (**a**) and GPN136 (**b**) on cell viability in primary human monocytes. NaF (250μg/ml) was used as a control (relative light unit, r.l.u). (**c**) Monocytes were treated with arachidonic acid (AA) in the absence or presence of 100μM GPN279 (white bars) and GPN136 (grey bars). The activity of COX1 was determined by measuring prostaglandin E2 (PGE2). Aspirin (10μM) was used as a control COX1 inhibitor (stripped bars). (**d**) Monocytes were incubated with lipopolysaccharide (LPS) (10U/ml) and arachidonic acid (AA) in the presence or absence of 100μM GPN279 (white bars) and GPN136 (grey bars). The activity of COX2 was determined by measuring PGE2. Diclofenac (10μM) was used as a control COX2 inhibitor (stripped bars). The data represent the means±SD of three independent experiments.

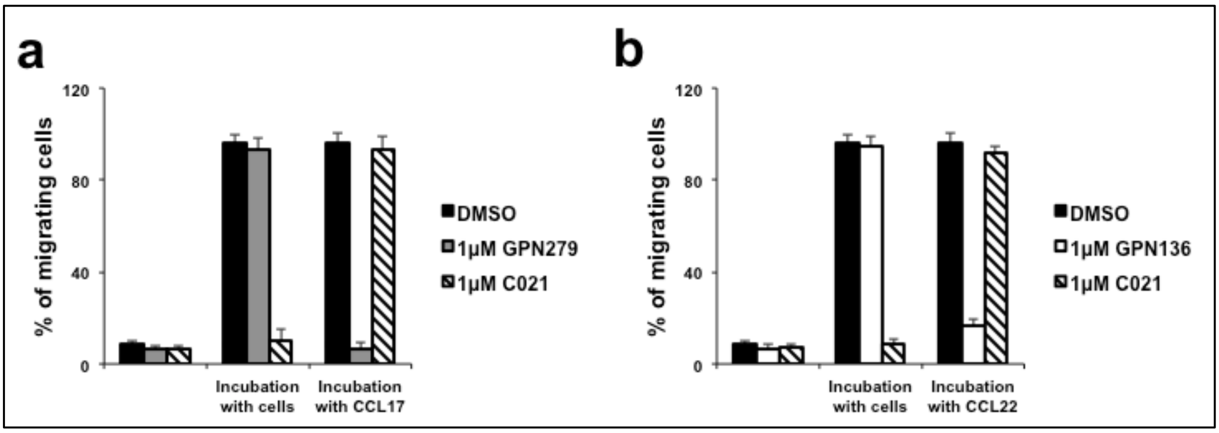

Supplementary Figure S4. Neutraligands inhibit CCL17 and CCL22- induced CCR4$^+$ HUT78 cell chemotaxis. Cell migration was carried out in RPMI-1640 medium containing 0.1% BSA using Transwell 96-well plates (Corning Costar, 5µm pore size, polycarbonate membranes). CCR4$^+$ HUT78 cells were re-suspended at 2×10^6/ml in assay buffer (RPMI 1640, 0.1% BSA) and placed in the upper wells of the Transwell plate. Lower wells contained the same medium diluted with DMSO or chemokine (3nM CCL17 or CCL22). In neutraligand inhibition studies, prior to their use, CCL17 (**a**) and CCL22 (**b**) were pre-incubated with DMSO (black bars), GPN279 (1µM, grey bars) and GPN136 (1µM, white bars), respectively, in the lower compartment. For characterization of the antagonist properties, the compounds were pre-incubated with the cells in the upper compartment. C-021 (1µM, stripped bars), the CCR4 receptor antagonist, was used as a control in both incubation protocols. Results are expressed as the percentage of cells that migrated to the lower chamber. Data represent the means±SD. Experiments were performed in triplicate.

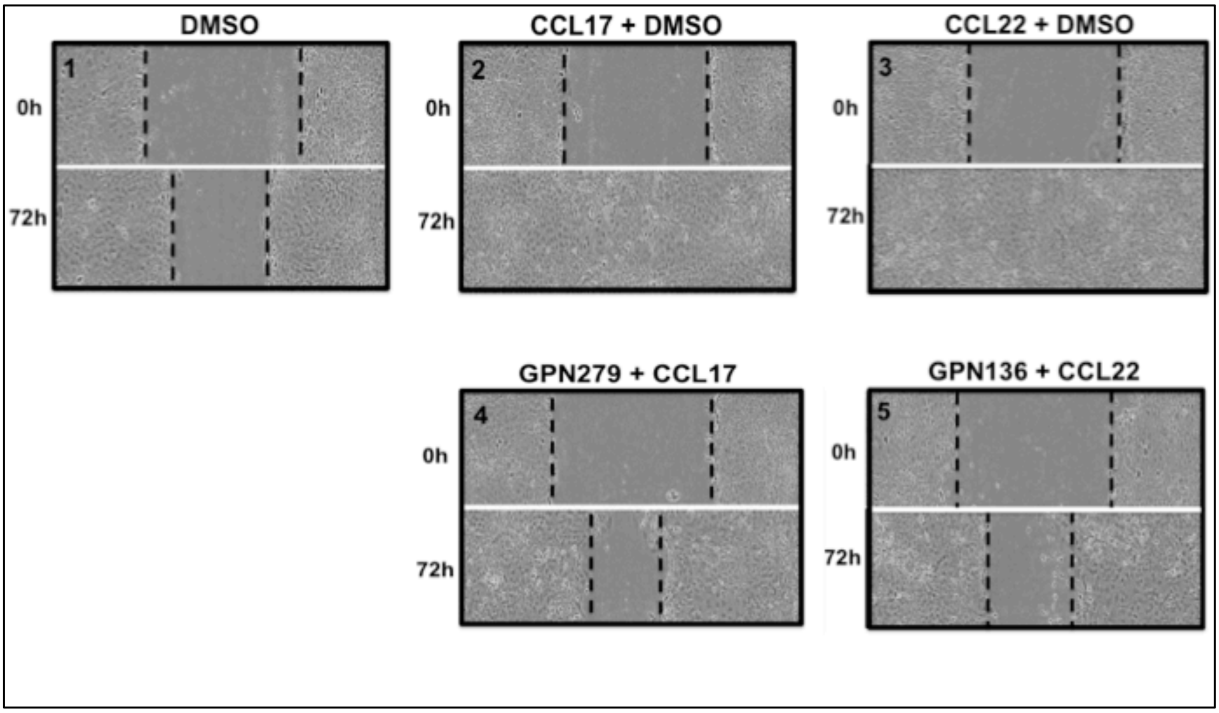

Supplementary Figure S5. Chemokine neutraligands inhibit HaCat cell migration induced by CCL17 or CCL22 in a scratch wound assay. Cells were treated with DMSO or the chemokine CCL17 or CCL22 (100ng/ml) in the presence or absence of the test compound (GPN279 or GPN136; 10µM). Cell migration within the wounded area was examined for 72h in the presence of mitomycin C. Representative images of the scratch wounds were taken with a ×10 objective from 0 to 72h. The experiments were performed in triplicate.

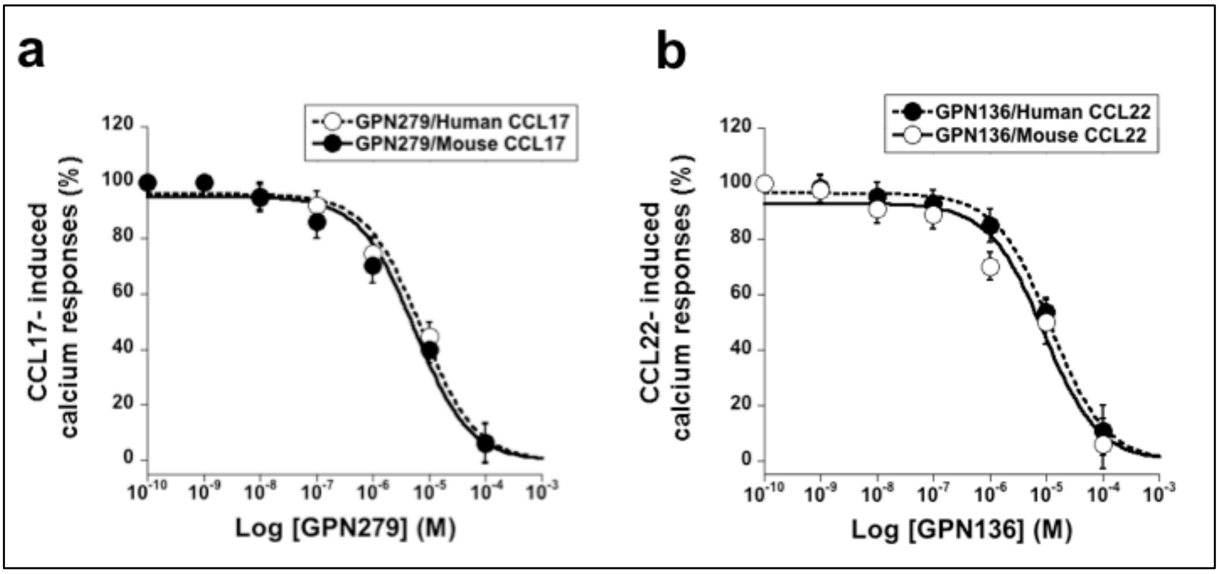

Supplementary Figure S6. (**a**) Inhibition of CCL17-induced calcium mobilization by GPN279 neutraligand. Human (○) or mouse (●) CCL17 (5nM) was preincubated with increasing concentrations of GPN279, 1h prior to addition to the CCR4$^+$ cells. (**b**) Inhibition of CCL22-induced calcium mobilization by GPN136 neutraligand. Human (●) or mouse (○) CCL22 (5nM) was preincubated with increasing concentrations of GPN136 (●), 1h prior to addition to CCR4$^+$ cells. CCR4$^+$Gqi5$^+$ HEK cells were loaded with the calcium-sensitive dye indo-1 AM; fluorescence intensity emitted at 401nm and 475nm in response to excitation at 355nm was recorded at 21°C.

Supplementary Tables

Supplementary Table S1. Small molecule screening data

Category	Parameter	Description
Assay	Type of assay	*In vitro* assay, $CCR4^{+}Gqi5^{+}$ HEK cells
	Target	CCL17 chemokine (TARC), CCL22 chemokine (MDC)
	Primary measurement	Detection of intracellular calcium responses induced by CCL17 or CCL22
	Key reagents	CCL17, CCL22, and Indo-1 AM calcium probe (Interchim)
	Assay protocol	The assay protocol is described in the "methods" section (calcium mobilization assay)
Library	Library size	480 compounds
	Library composition	Natural products
	Source	Plants, fungi, algae
	Additional comments	Storage of the library at -20°C, additional information could be found on the web site of GreenPharma (http://www.greenpharma.com)
Screen	Format	96-well plates
	Concentration(s) tested	2µg/ml compound, 1%DMSO
	Plate controls	Chemokine + DMSO, buffer + DMSO, chemokine + receptor antagonist (C-021), buffer + receptor antagonist (C-021)
	Detection instrument and software	FlexStation 3 (Molecular Device)
	Assay validation/QC	Z' score (>0.5)
	Normalization	Digitonin
Post-HTS analysis	Additional assay(s)	Physicochemical parameters (solubility, stability, ...), Tryptophan fluorescence, selectivity, *in vitro* toxicity, chemotaxis, receptor endocytosis, cyclooxygenase and phosphodiesterase inhibition, scratch wound assay, *in vivo* testing
	Confirmation of hit purity and structure	Compounds were resynthetized and repurchased (Ambinter.com/GreenPharma.com) Structure and purity were verified analytically

Supplementary Table S2. Inhibition of calcium responses induced by CCL17 and CCL22 by compounds (5µM). Compounds 2 to 5 bear structural similarity with GPN279 (compound 1), and compounds 7 to 14 are structural analogues to GPN136 (compound 6). Inhibition is expressed as percent. Theophylline (compound 2, absence of isoprene group), caffeine (compound 3) and IBMX (compound 5, 3-isobutyl-1-methyl xanthine, a phosphodiesterase inhibitor) had no effect on calcium responses induced by CCL17 by contrast to GPN279 (42.0±4.0% inhibition). The percentage of inhibition of the calcium responses induced by CCL22 was 37.0±6.0% and 40.0±5.0% for GPS008505 (compound 8) and GPS008506 (compound 9), respectively. These two enantiomeric molecules had almost the same efficacy as the racemic GPN136, thereby demonstrating that the asymmetric carbon atom is not critical to the activity of GPN136. In addition, the dimeric GPN136 compound, GPS008504 (compound 7), had a low activity on CCL22 (10.0±5.0% inhibition) that may be due to the steric hindrance of the compound. GPS008507 (compound 10) and GPS008508 (compound 11) (which bear no hydroxyl group on the phenyl) had no effect on CCL22-induced calcium responses, suggesting the presence of an hydroxyl group is important in maintaining the activity on CCL22. Also, aspirin (compound 13) had no activity on CCL22 (0.0% inhibition), which may be related to the absence of an hydroxyl group. In addition, the salicylate GPN001207 (compound 14) had no inhibitory effect of CCL22-induced calcium responses, indicating the importance of the glycerol chain moiety of GPN136. Cpd: compound.

Cpd	Reference	Structure	Inhibition of CCL17 calcium responses (%)	Inhibition of CCL22 calcium responses (%)
1	GPN279	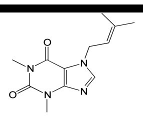	42.0± 4.0	0.0
2	Theophylline	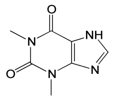	0.0	0.0
3	Caffeine	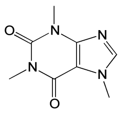	0.0	0.0
4	GPN062	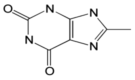	0.0	0.0
5	IBMX	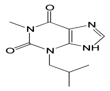	0.0	0.0
6	GPN136	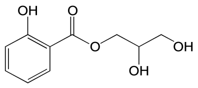	0.0	35.0± 8.0
7	GPS008504	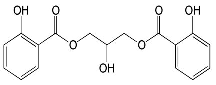	0.0	10.0± 5.0
8	GPS008505	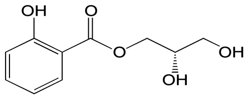	0.0	37.0± 6.0
9	GPS008506	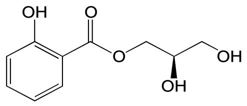	0.0	40.0± 5.0
10	GPS008507	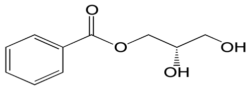	0.0	0.0
11	GPS008508	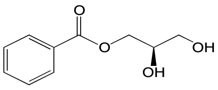	0.0	0.0
13	Acetylsalicylic acid	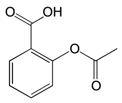	0.0	0.0
14	GPN0001207	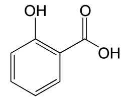	0.0	0.0
